# Modeling potential responses to smallpox as a bioterrorist weapon.

**DOI:** 10.3201/eid0706.010607

**Published:** 2001

**Authors:** M I Meltzer, I Damon, J W LeDuc, J D Millar

**Affiliations:** Centers for Disease Control and Prevention, Atalnta, Georgia 30333, USA.qzm4@cdc.gov

## Abstract

We constructed a mathematical model to describe the spread of smallpox after a deliberate release of the virus. Assuming 100 persons initially infected and 3 persons infected per infectious person, quarantine alone could stop disease transmission but would require a minimum daily removal rate of 50% of those with overt symptoms. Vaccination would stop the outbreak within 365 days after release only if disease transmission were reduced to <0.85 persons infected per infectious person. A combined vaccination and quarantine campaign could stop an outbreak if a daily quarantine rate of 25% were achieved and vaccination reduced smallpox transmission by > or = 33%. In such a scenario, approximately 4,200 cases would occur and 365 days would be needed to stop the outbreak. Historical data indicate that a median of 2,155 smallpox vaccine doses per case were given to stop outbreaks, implying that a stockpile of 40 million doses should be adequate.


### Modeling Potential Responses to Smallpox as a Bioterrorist Weapon

**Martin I. Meltzer,* Inger Damon,* James W. LeDuc,* and J. Donald Millar†**
*Centers for Disease Control and Prevention, Atlanta, Georgia, USA; and
†Don Millar & Associates, Inc., Atlanta, Georgia, USA

We constructed a mathematical model to describe the spread of smallpox after a deliberate release of the virus. Assuming 100 persons initially infected and 3 persons infected per infectious person, quarantine alone could stop disease transmission but would require a minimum daily removal rate of 50% of those with overt symptoms. Vaccination would stop the outbreak within 365 days after release only if disease transmission were reduced to $\geq$0.85 persons infected per infectious person. A combined vaccination and quarantine campaign could stop an outbreak if a daily quarantine rate of 25% were achieved and vaccination reduced smallpox transmission by $\geq$33%. In such a scenario, approximately 4,200 cases would occur and 365 days would be needed to stop the outbreak. Historical data indicate that a median of 2,155 smallpox vaccine doses per case were given to stop outbreaks, implying that a stockpile of 40 million doses should be adequate.

Recent papers have speculated about the use of smallpox as a biological weapon (1-5). If we assume such a risk, there is concern about the need for preparations to limit and prevent the spread of smallpox after a deliberate release of the virus. Studies of smallpox control and eradication efforts (6-8) identified two available types of interventions: vaccination of those at risk from infection, quarantine, or both. Some studies have provided estimates of the potential numbers that could be infected (1,3,5) and the number of vaccine doses that should be stockpiled (5); however, they did not provide details of how these estimates were calculated. Further, none of these articles examined how quarantine of infected persons may help halt transmission of smallpox.

Crucial questions that remained unanswered include—How can we calculate the number of doses of smallpox vaccine to be stockpiled? Can quarantine contribute to control efforts? How effective does quarantine have to be to reduce transmission? We present a mathematical model that helps answer these and other questions.

### Methods

We constructed a mathematical model to meet the following objectives: 1) describe the spread of smallpox through a susceptible population, calculating daily (new-onset) and cumulative cases; 2) readily accommodate changes in input values, such as the number of persons infected per infectious person (i.e., rate of transmission) and the number of persons initially infected; 3) examine the impact of quarantine and vaccination, alone and in combination, on the spread of smallpox; and 4) estimate the number of doses of smallpox vaccine that should be stockpiled as part of readiness plans.

Address for correspondence: Martin I. Meltzer, Centers for Disease Control and Prevention; Mailstop D-59; 1600 Clifton Rd., Atlanta, GA 30333, USA; fax: 404-371-5445; e-mail: qzm4@cdc.gov

Despite numerous reports of mathematical models of infectious diseases (9-14), few such models describe the spread of smallpox. Frauenthal (15) addressed the question of optimal level of smallpox vaccination. We constructed a Markov chain model (16) to describe the spread of smallpox through a susceptible population (objective 1), using a computer-based spreadsheet program (Excel97, Microsoft, Redmond, WA). The model describes four disease stages: incubating, prodromal, overtly symptomatic, and no longer infectious (Figure 1). The term "prodromal" indicates the preeruptive stage.[1] "Overtly symptomatic" refers to the period of disease when a rash or similar symptoms can be readily noted by even an untrained observer.[2] For each day after the release, the model calculates both the number of new cases and the cumulative total.

In the model, an infected person can only progress, from incubating to prodromal to overtly symptomatic, and cannot revert. The duration in days of a given disease stage is controlled by a probability function (Figure 2).

### Probable Durations of Each Disease Stage

When smallpox was endemic in human populations, the incubation period was often difficult to measure because many patients were exposed over several days (7,8). Fenner et al. (7) reviewed and summarized three reports in which the incubation period was calculated for 255 cases of variola major smallpox (the "classic" form). Just over 70% of these cases incubated 9 to 13 days, with an average of 11.5 days (range 7 to 19 days; median approximately 11 days; 5th

[1]Others have suggested that the terms "preeruptive" or "initial" are more descriptively accurate of this stage (6). However, because "prodromal" is used in many standard textbooks (7,8,17), we will use this term.
[2]Prodromal rashes have been recorded, but they were considered to be uncommon occurrences, ". . . not more than 1 in 10." (17).

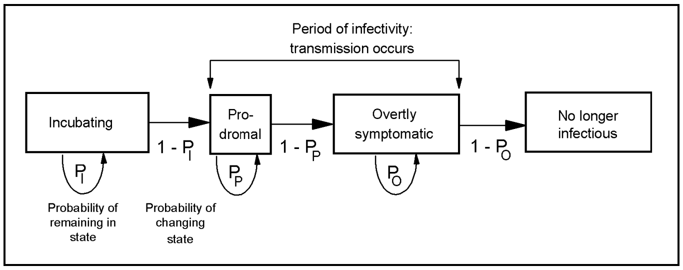

Figure 1. Schematic of the Markov-chain model used to model the movement of a person infected with smallpox through the four stages of disease. $P_I$ = probability of remaining in the incubating stage; $P_P$ = probability of remaining in the prodromal stage; and $P_O$ = probability of remaining in the overtly symptomatic stage. For each stage, the probabilities of remaining in that stage ($P_I$,$P_P$,$P_O$) are determined by a daily probability (Figure 2). Patients who have reached the fourth and final stage (no longer infectious) effectively drop out of the model. The "overtly symptomatic" stage refers to the period of disease when a person has a rash or similar symptoms that even an untrained observer can readily note. During the period of infectivity, the average number of persons infected per infectious patient is preset by the researchers. The days when transmissions occur are determined by a probability function.

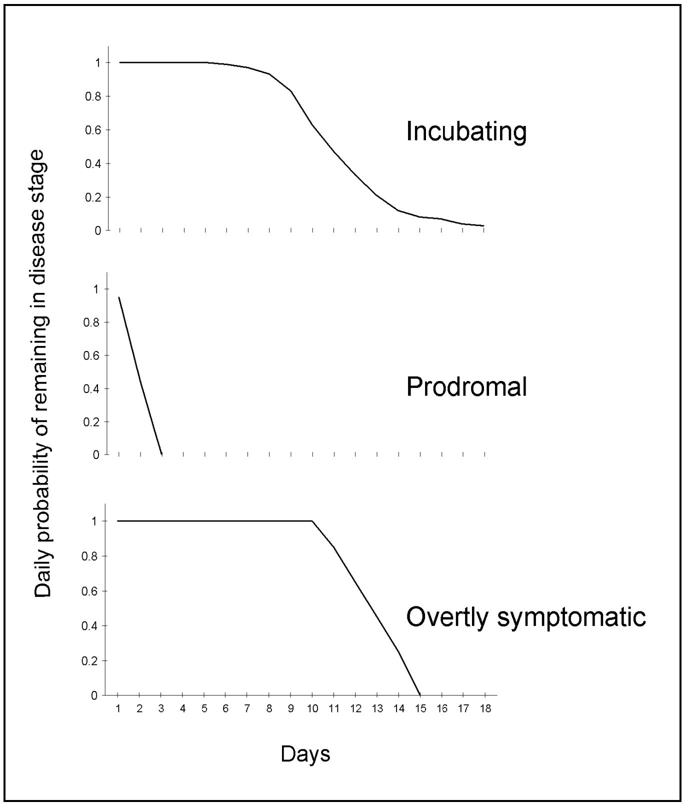

Figure 2. Probability functions associated with remaining in three smallpox disease stages. These reverse cumulative probability functions describe the probability on any defined day of a patient remaining in a disease stage during the next day. On any given day, the probability of moving from one stage to the next is 1 minus the probability of remaining in the stage.

percentile 8 days; and 95th percentile 14 days). Others have observed similar lengths of incubation. For example, by examining the time between onset and "brief and only possible contact with a known case," Singh (18) determined the possible length of incubation of six cases of smallpox (mean 11 days; median 12 days). Rao (6) used data from 50 first-generation cases to determine that the mean "fever-to-fever" (i.e., onset of fever to onset of fever) interval was 16 days (range 12 to 21 days for 80% of cases).

Using data from 115 cases in Europe (19), we constructed a reverse cumulative probability function to describe the probability of a person on a given day remaining in the state of incubation for the next day (Figure 2). The calculated mean was 11.7 incubating days (median approximately 11 days; 5th percentile 8 days; and 95th percentile 17 days). The function used can be altered to reflect other data sets or hypothesized functions. Further, the model can accept different transition probability functions for each day in the model.

The duration of the prodromal stage is variable and depends in part on the ability of the physician or patient to detect the first lesion (6). The onset of rash (the overtly symptomatic stage) typically occurs 48 to 72 hours after onset of fever, although some types of smallpox may have a prolonged prodromal stage of 4 to 6 days (6). Fenner et al. reviewed several data sources and used temperature data to report that the prodromal stage lasts an average of 3 days (7). Beyond these descriptions of the average or typical course of disease, no data are readily available documenting the probabilities associated with a longer prodromal stage (e.g., frequency data linking number of patients to number of days in the prodromal stage). Thus, we assumed a linear decline in the daily probability of remaining in the prodromal stage (Figure 2). The probabilities decline from 0.95 at the end of day 1 (i.e., a 95% chance that the patient will be in prodromal stage for another day) to 0.00 at the end of day 3 (i.e., absolute certainty that the prodromal stage will not last beyond day 3).

The average total time of illness (i.e., having some symptoms) is given in Fenner et al. (7) as 21 days, with scabbing on day 19. Allowing up to 3 days for the prodromal period (Figure 2) leaves an average of 16 days in the overtly symptomatic period in which a patient can infect others. Although scabs may contain infectious amounts of smallpox virus after the patient has fully recovered, we assumed that after scabbing, neither the patient nor the scabs will pose a substantial source of infection. The exact duration of illness is somewhat moot, as the likelihood of transmission declines after the first few days of overt symptoms. Thus, after some period, a person who is overtly symptomatic has a low probability of infecting a susceptible person. We assumed a probability of 1.00 (i.e., absolute certainty) of remaining the next day in the overtly symptomatic stage for the first 10 days in the stage. Including the prodromal stage, this corresponds to 12-15 days of illness (Figure 2). After 10 days, a patient's daily probability of remaining in the stage decreases linearly, so that 15 days after onset of symptoms the probability of remaining the next day in this stage is 0.00 (Figure 2). That is, after a maximum of 16 days in the overtly symptomatic stage, all patients will have progressed to the "no longer infectious" stage. Patients who have reached the fourth and final stage (no longer infectious) effectively drop out of the model. These probability functions can readily be changed (objective 2).

### Likelihood of Smallpox Transmission

Also described by a probability function is the likelihood of smallpox transmission during the infectious period. For a variety of reasons, the probability of transmission is likely to change during the period when an infected person is infectious. For example, persons with a high fever during the first

2 days of the prodromal stage (Figure 2) may voluntarily confine themselves to quarters, possibly limiting their opportunities to infect others. Limited data are available regarding changes in the probability of when an infection is transmitted, but Mack (19) and Rao (6) provide a time series of data involving 23 and 60 patients, respectively. Both data sets suggest that transmission is less likely during the prodromal stage (the first 3 days when a person is symptomatic) and that the probability of transmission is greatest between days 3 and 6 after a patient becomes infectious (Figure 3). This period is equivalent to the first to third days of onset of rash (overt symptoms). Both data sets (6,19) indicate that 70% to 80% of transmission is likely to occur in the first 9 days of the symptomatic period, and 90% of all transmission will have occurred in 10 to 13 days (Figure 3). In other words, by day 6 of overt symptoms (rash), approximately 75% of transmissions will have occurred, with 90% occurring within 7 to 10 days. For the model, we used the data from Mack (19) to describe the probabilities of when transmission will occur, from infectious to newly infected (Figure 3). Other data sets and probability functions can readily be substituted.

### Existing Immunity and Community Size

For simplicity, we assumed an unlimited supply of susceptible persons,[3] so that disease transmission will not be halted because of lack of susceptible persons. Although this scenario is unrealistic for modeling the natural spread of an infectious disease, it may be realistic for considering the initial spread of an infectious disease after deliberate infection of a small number of persons in a population with a relatively large proportion who are susceptible.

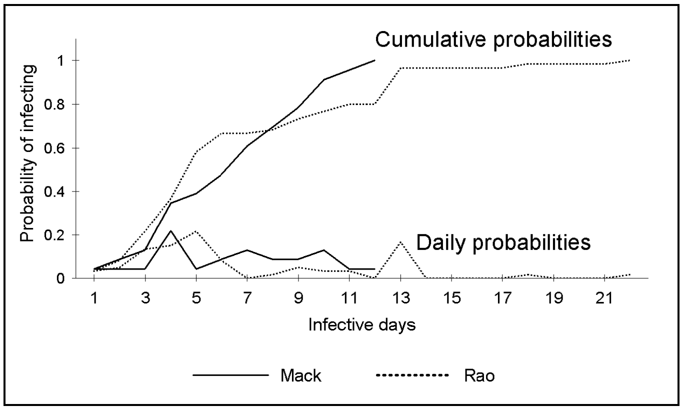

Figure 3. Daily and cumulative probabilities determining when an infectious person infects another person with smallpox (6,19). Day 1 of the infectious period is the first day of the prodromal stage. That is, we have interpreted the source data to reflect the assumption that no spread of infection can occur while an infected person is in the incubating stage.

Another variable that can alter the transmission rate and persistence of disease is size of community. Smith (22) summarized data evaluating the link between community size and spread of some infectious diseases and found that the larger the community, the higher the rate of transmission. This observation was found to be true for measles, scarlet fever, diphtheria, and whooping cough (pertussis), but smallpox was not analyzed (22-24). Arita et al. (25) found a correlation between increasing density of smallpox-susceptible persons and the persistence of smallpox within a population but did not estimate the relationship between susceptible population density and transmission rate. Our model allows for the impact of different densities of susceptible persons by adjusting the average transmission rate.

### Numbers Initially Infected and Rate of Transmission

Based on Henderson's comment that an outbreak of smallpox ". . . in which as few as 100 people were infected would quickly tax the resources of any community" (1), we initially assumed that 100 persons would be effectively exposed, infected, and become infectious. We set the average transmission rate at 3, which is notably higher than most historical averages. (A mathematical review of the transmission of smallpox appears in Appendix I, available at URL: http://www.cdc.gov/ncidod/eid/vol7no6/meltzer_appendix1.htm). We define the term "transmission rate" as the number of persons infected per infectious person, rather than the number of persons infected during a standardized unit of time. During sensitivity analyses, we altered both the number of persons initially infected and the rate of transmission.

### Modeling the Effects of Potential Interventions

We examined the effect of quarantine and vaccination, alone and in combination (objective 3). Quarantine was modeled by removing daily a fixed proportion of a cohort of infectious persons, starting on the day that they become overtly symptomatic. For example, we assumed that 50% of all persons with rashes on day 1 of the overtly symptomatic period would be successfully quarantined and not infect anyone else. Fifty percent of those who missed quarantine on day 1 of rash would be quarantined on day 2.[4] This proportionate reduction would continue for the duration of time that persons are likely to infect others. The model also calculated the number of infectious persons needed to be quarantined under a given scenario.

For a vaccination-only strategy to stop transmission, sufficient susceptible persons must be effectively vaccinated so that the number of persons infected per infectious person is less than 1. We thus evaluated how long it would take to stop an outbreak if the level of transmission were reduced to 0.99 persons infected per infectious person. We also calcu-

[3]The United States stopped routine vaccination of the civilian population in 1972 (5). In July 1998 in the United States, there were approximately 109.9 million persons ≤30 years of age, representing 41% of the total resident population (20). Most of these people have not been vaccinated against smallpox. In addition, the immunologic status of those who were vaccinated >30 years ago must be considered. Historical data indicate that vaccination 20 to 30 years ago may not protect against infection but will often protect against death (8,21). No reports, however, define the probability of such persons' transmitting the disease to susceptible persons. Faced with such uncertainty, we chose the simplest approach of assuming an unlimited supply of susceptible persons.

[4]At a 50% daily removal rate, a cohort of all those beginning the first day of overt symptoms is entirely removed in 7 days (8 to 10 days postincubation), with 90% removed in 4 days after they enter the overtly symptomatic period. At a 25% daily removal rate, a cohort is entirely removed 17 days after entering the overtly symptomatic period (18 to 20 days postincubation), with 90% removed in 9 days after entering the overtly symptomatic period. The calculated numbers of those quarantined relate only to those who are infectious (i.e., overtly symptomatic). The model does not take into account those who might also be quarantined along with the infectious persons, such as unvaccinated household contacts and other exposed persons.

lated the smallest vaccine-induced reduction in transmission required to stop the outbreak within 365 days postrelease. This calculation was done by an iterative process in which the rate of transmission was reduced until the number of new cases per day reached approximately zero 365 days after release. To estimate the impact of vaccination, we assumed that a vaccination campaign would immediately reduce the risk of transmission, and we did not model the time required from vaccination to effective vaccine-derived immunity. This assumption may overstate the impact of vaccination, particularly in terms of how quickly a vaccination campaign could stop an outbreak.

Lane and Millar estimated that continuing routine childhood immunization against smallpox in the United States from 1969 to 2000 would cause 210 vaccine-related deaths (26). That calculation was made before the population included substantial numbers of immunocompromised persons (e.g., HIV- or cancer therapy-induced immune suppression). Because of the potential for adverse vaccine-related side effects,[5] it may be prudent to attempt to limit the number of persons vaccinated. We therefore calculated the impact of limiting the numbers vaccinated so that transmission would be reduced by just 25%, from 3 to 2.25 persons infected per infectious person, combined with a daily quarantine rate of 25%. We also calculated, by an iterative process, the smallest vaccine-induced reduction in transmission required to stop the outbreak within 365 days postrelease when combined with a daily quarantine rate of 25%.

### Start of Interventions

We considered the effect of starting large-scale, coordinated interventions on days 25, 30, and 45 postrelease, assuming release on day 1. Twenty-five days assumes 15 days for the first signs of overt symptoms (Figure 2), 2 days for initial clinical diagnosis, 1 day for specimen transport, 3 days for laboratory confirmation, and 4 days to mobilize and begin appropriate large-scale interventions.[6] Although interventions may begin on a small scale earlier than day 25, in the model the term “start date of interventions” refers to the date when a full-scale and comprehensive intervention begins (i.e., the model does not allow for a gradual increase of intensity in interventions). If we assume that an average of 15 days will be needed for those infected to become infectious (Figure 2), 30 days represents the time when the first generation of cases (those infected by the index cases) will begin to show overt symptoms. Forty-five days represents the time needed for the second generation of cases (those infected by the first generation) to show overt symptoms.

### Numbers Vaccinated per Case: Stockpile Issues

To determine the number of persons that must be vaccinated, we searched for reports of successfully contained smallpox outbreaks in which both the number of cases and the number of doses of vaccine administered were recorded. These data allowed us to assemble a data set of doses used per case, which was then fitted to probability distributions by using specialized software (Bestfit, Palisade Corp, Newfield, NY). The probability distribution that gave the “best fit,” judged by standard tests (chi square, Kolmogorov-Smirnov, Anderson-Darling), provided the mean and median number of doses historically used per case of smallpox, as well as confidence intervals (e.g., 95th, 90th, and 10th percentiles). We then estimated the total number of vaccine doses that should be stockpiled by multiplying the estimated doses per case by the number of cases estimated by the Markov chain model (objective 4).

### Other Potential Interventions

We did not consider other potential preparations, such as routine mass immunizations against smallpox. Reasons for this exclusion include uncertainties about cost, vaccine safety, duration of vaccine efficacy, and the probability of such an event.

### Sensitivity Analyses

We examined the effect on the number of daily and total cases when the number initially infected was changed from 100 to 1,000 and the transmission rate was decreased to 2 or increased to 5 persons infected per infectious person. We also used the model to determine the minimum level of interventions needed to ensure that transmission stopped by given target dates. We chose 75, 150, and 225 days postrelease as the examples of target dates, representing 5, 10, and 15 generations of smallpox, respectively. The minimum levels of intervention needed to achieve these targets were determined by an iterative process, altering the level of the intervention(s) until the number of new cases per day reached zero on each target date.

### Results

### Effect of Transmission Rate and Numbers Initially Infected

We calculated the hypothetical effect of allowing smallpox to spread without intervention, assuming an unlimited supply of smallpox-susceptible persons. The data demonstrate that the most important mathematical variable is the assumed rate of transmission. For a given number of persons initially infected, doubling the number infected per infectious person causes a massive increase (greater than 2 orders of magnitude) in the cumulative total cases at 365 days (Table 1).

### Effect of Intervention: Quarantine Only

A quarantine-only program can stop an outbreak of smallpox, but it takes a daily removal rate of at least 50% to ensure that disease transmission will cease. At a quarantine rate of 50% starting on day 30 postrelease, the daily number of new cases would peak at approximately 50 cases per day, with no new cases on day 240 and a cumulative total of approximately 2,300 cases (Figure 4). If 50% quarantine began 5 days earlier, on day 25 postrelease, the total cases would be approximately 1,750 and the maximum number of daily new cases would be 20 per day (Figure 4). A 15-day delay in starting quarantine programs, to day 45 postrelease, results in approximately 6,800 total cases and a maximum of almost 120 new cases daily (Figure 4).

[5] The number, severity, and cost of vaccine-induced side effects is the subject for a separate paper.
[6] Allowing 3 days for laboratory confirmation assumes that virus loads in clinical specimens may be insufficient to allow use of rapid assays and confirmation must await the results of a culture-based assay, which takes approximately 72 hours. Rapid laboratory confirmation, within 24 hours, is possible.

Table 1. Estimates of cumulative total smallpox cases after 365 days with no intervention

| No. initially infected[a] | No. infected per infectious person[b] | Cumulative total no. of smallpox cases, days postrelease[c] | | | |
|---|---|---|---|---|---|
| | | 30 days | 90 days | 180 days | 365 days |
| 10 | 1.5 | 31 | 214 | 2,190 | 224 thousand |
| 10 | 3.0 | 64 | 4,478 | 2.2 million | 774 billion |
| 1,000 | 1.5 | 3,094 | 21,372 | 219,006 | 22 million |
| 1,000 | 3.0 | 6,387 | 447,794 | 222 million | 77 trillion |

[a] Number initially infected refers to those who are exposed during a release so that they subsequently become infectious to others. This scenario excludes those who are exposed but either do not become ill (i.e., are immune or are not exposed to an infectious dose) or do not become infectious (residual immunity from prior vaccination may be sufficient to prevent onward transmission).
[b] The number of persons infected per infectious person is the transmission rate.
[c] Assumes an unlimited supply of smallpox-susceptible persons.

### Effect of Intervention: Vaccination Only

A vaccination-only program that reduces the rate of transmission to 0.99 persons infected per infectious person will eventually stop an outbreak, but not within 365 days postrelease, even if it is begun on day 25 postrelease (Figure 5). To stop the outbreak by day 365 postrelease, a vaccination campaign starting on day 30 must reduce transmission to approximately 0.85 persons infected per infectious person (Figure 5), resulting in a cumulative total of 2,857 cases. If the same intervention were started on day 25 postrelease, the cumulative total would decline to 2,125 cases. Delaying the start of the intervention to day 45 postrelease would result in 3 new cases per day and a cumulative total of 8,347 casesonday365.

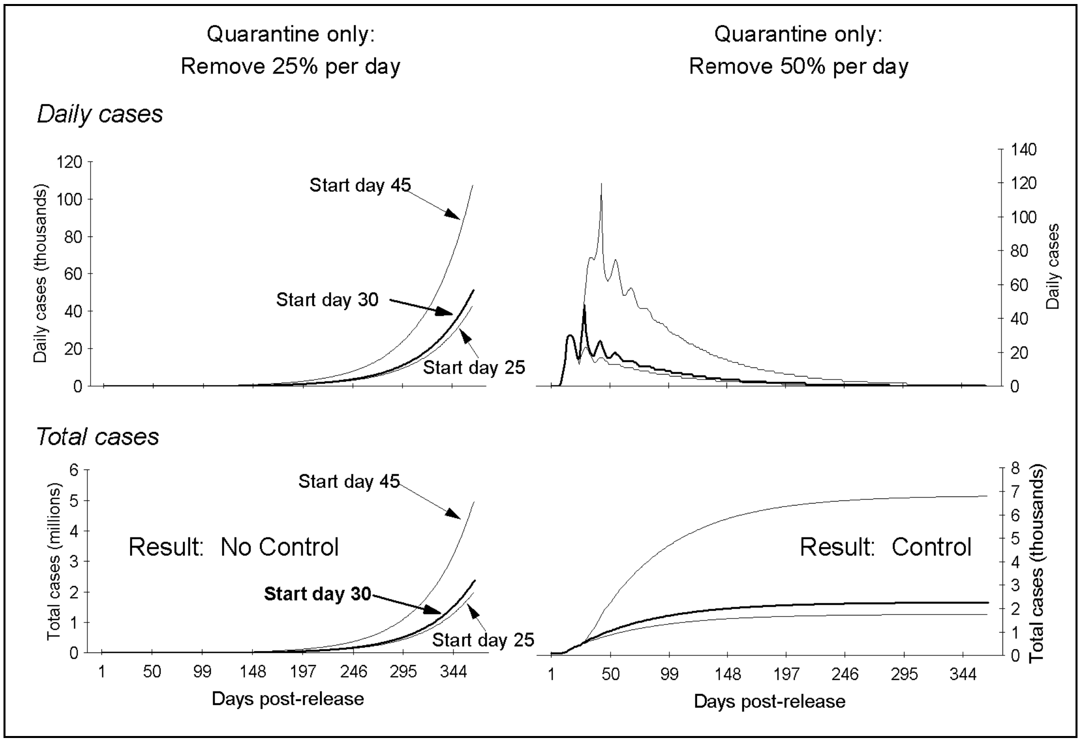

Figure 4. Daily and total cases of smallpox after quarantining infectious persons at two daily rates and three postrelease start dates. The graphs demonstrate that if quarantine is the only intervention used, a daily removal rate of ≥ 50% is needed to stop transmission within 365 days postrelease. At a 25% daily removal rate of infectious persons by quarantine, a cohort of all those entering the first day of overt symptoms (i.e., rash) is entirely removed within 17 days (18 to 20 days postincubation) after the first day of overt symptoms, with 90% removed within 9 days. At a 50% daily removal of infectious persons by quarantine, a cohort of all those entering their first day of overt symptoms (i.e., rash) is entirely removed within 7 days (8 to 10 days postincubation) after the first day of overt symptoms, with 90% removed within 4 days. The daily rate of removal (quarantine) relates only to the removal of those who are infectious (i.e., overtly symptomatic). The rate does not include any persons who may be quarantined along with overtly symptomatic patients, such as unvaccinated household contacts. Data generated by assuming 100 persons initially infected and a transmission rate of 3 persons infected per infectious person. For clarity, the graphs of daily cases do not include the assumed 100 initially infected persons. The graphs of total cases include the 100 initially infected

### Effect of Intervention: Quarantine and Vaccination

When combined with a quarantine rate of 25%, to stop transmission by day 365 postrelease, vaccination has to effectively reduce the rate of transmission by at least 33%, from 3 persons infected to 2 persons infected per infectious person (Figure 6). Although transmission will be halted,[7] the total number of cases would be approximately 4,200, which is 82% greater than the total if a 50% daily reduction quarantine-only program is assumed (Figure 4). Starting on day 25 postrelease reduces the total number of cases to approximately 3,200 (Figure 6). Delaying the start of a combined intervention to day 45 postrelease increases the total number of cases to approximately 12,400.

[7] Even by reducing transmission from 3 to 2 persons per infectious person and quarantining infectious persons at a rate of 25% per day, the number of new cases at day 365 is 3, not zero (i.e., transmission is not quite completely stopped) (Figure 6). For transmission to cease completely, vaccination must either achieve a 38% reduction in transmission to 1.85 cases per infectious person (assuming a daily quarantine rate of 25%), or quarantine must achieve a 29% daily reduction in the number of infectious persons (assuming vaccination reduces transmission by 33%).

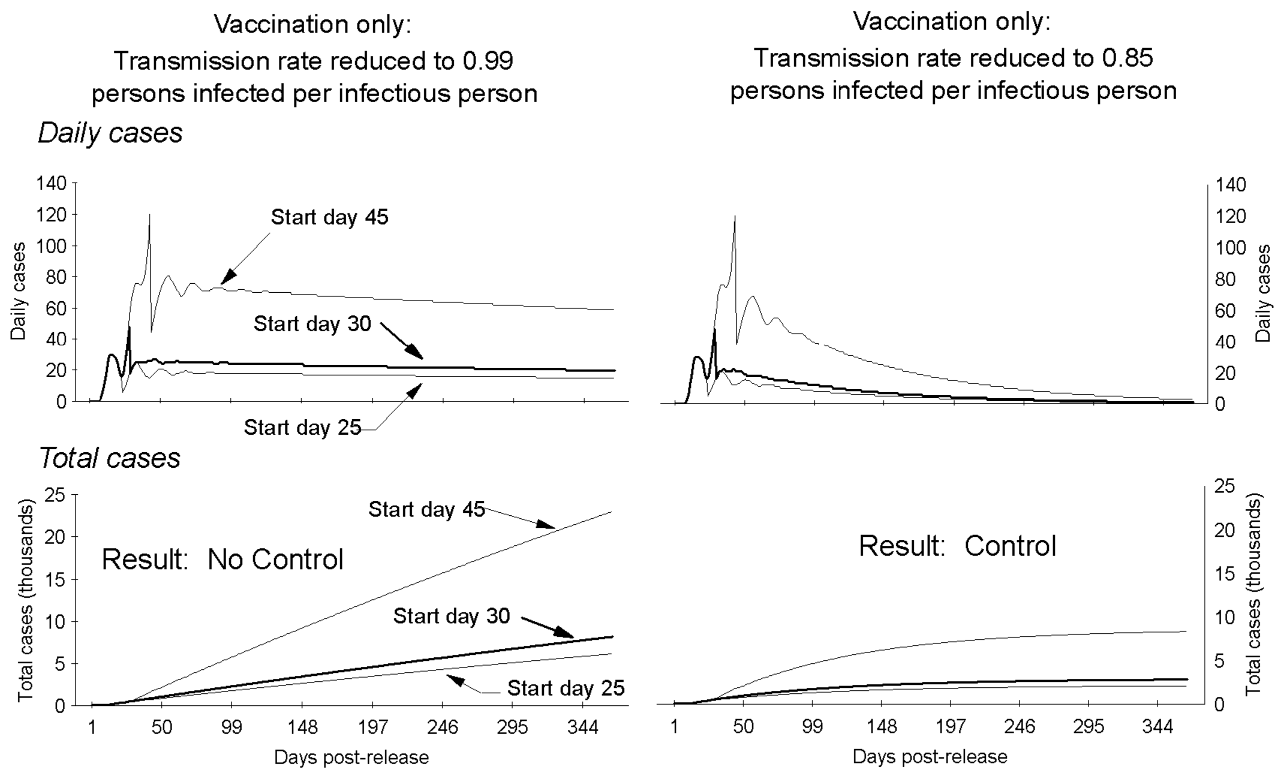

Figure 5. Daily and total cases of smallpox for two vaccine-induced rates of transmission and three postrelease start dates. The graphs show that, while reducing the transmission rate to 0.99 persons infected per infectious person reduces the daily number of cases over the period studied, vaccination must reduce the rate of transmission to 0.85 persons infected per infectious person to stop the outbreak within 365 days postrelease. Data were generated by assuming 100 initially infected persons and an initial transmission rate of 3 persons infected per infectious person. For clarity, the graphs of daily cases do not include the assumed 100 initially infected. The graphs of total cases include those initially infected.

### Effect of Intervention: Number of Infectious Persons Quarantined

With a quarantine-only intervention of 50% daily rate of removal, starting on day 30 postrelease, the peak number of daily removals is 69 infectious persons, occurring on day 30 (start day) with a cumulative total of 2,166 infectious persons quarantined. With a combination of a 33% vaccine-induced reduction in transmission and a 25% daily removal quarantine program, the peak number of daily removals is 34 (start day 30), but the cumulative total that must be quarantined is approximately 3,970 infectious persons.

### Sensitivity Analyses: Effect of Changing Input Values

Reducing the transmission rate to two results in a quarantine-only program with a 25% daily removal rate almost stopping transmission (Table 2). Delaying the start of such an intervention to day 45 but combining it with a vaccination campaign, which reduced transmission by 33%, would halt the outbreak by Day 365 (Table 2). For the same intervention start date, increasing the assumed transmission rate from 2 to 5 persons infected per infectious person does not proportionately increase the cumulative total number of cases at day 365. Even with a quarantine rate of 25% removal per day, assuming that vaccination concurrently reduces transmission by 66%, the cumulative total number of cases on day 365 is 19,821 (Table 2). For any given scenario, increasing the number initially infected from 100 to 1,000 increases both the cumulative totals and the daily number of new cases at day 365 by a factor of 10 (Table 2). Similarly, reducing the number of those initially infected from 100 to 10 would cause a proportionate reduction in both cumulative totals and daily numbers (data not shown; additional results in Appendix II, available at URL: http://www.cdc.gov/ncidod/eid/vol7no6/meltzer_appendix2.htm).

### Sensitivity Analyses: Minimum Levels of Intervention to Achieve Target Days

The earlier the target date for stopping an outbreak, the larger the minimum vaccine-induced reduction in transmission needed to achieve zero transmission (i.e., outbreak stopped). For example, assuming a transmission rate of 3 and a 25% daily removal rate, a target date of day 225 requires a 45.2% vaccine-induced reduction in transmission to 1.65 persons infected per infectious person (Table 3). Reducing the target date to day 75 requires a 76.7% vaccine-induced reduction in transmission to 0.70 persons infected per infectious person (Table 3). Again, delay in starting interventions makes it notably more difficult to stop an outbreak by a given target date. For example, to achieve a target date of day 75 with a 50% daily removal rate, starting interventions on day 45 requires a vaccine-induced reduction in transmission of 81.2%, to 0.57 persons

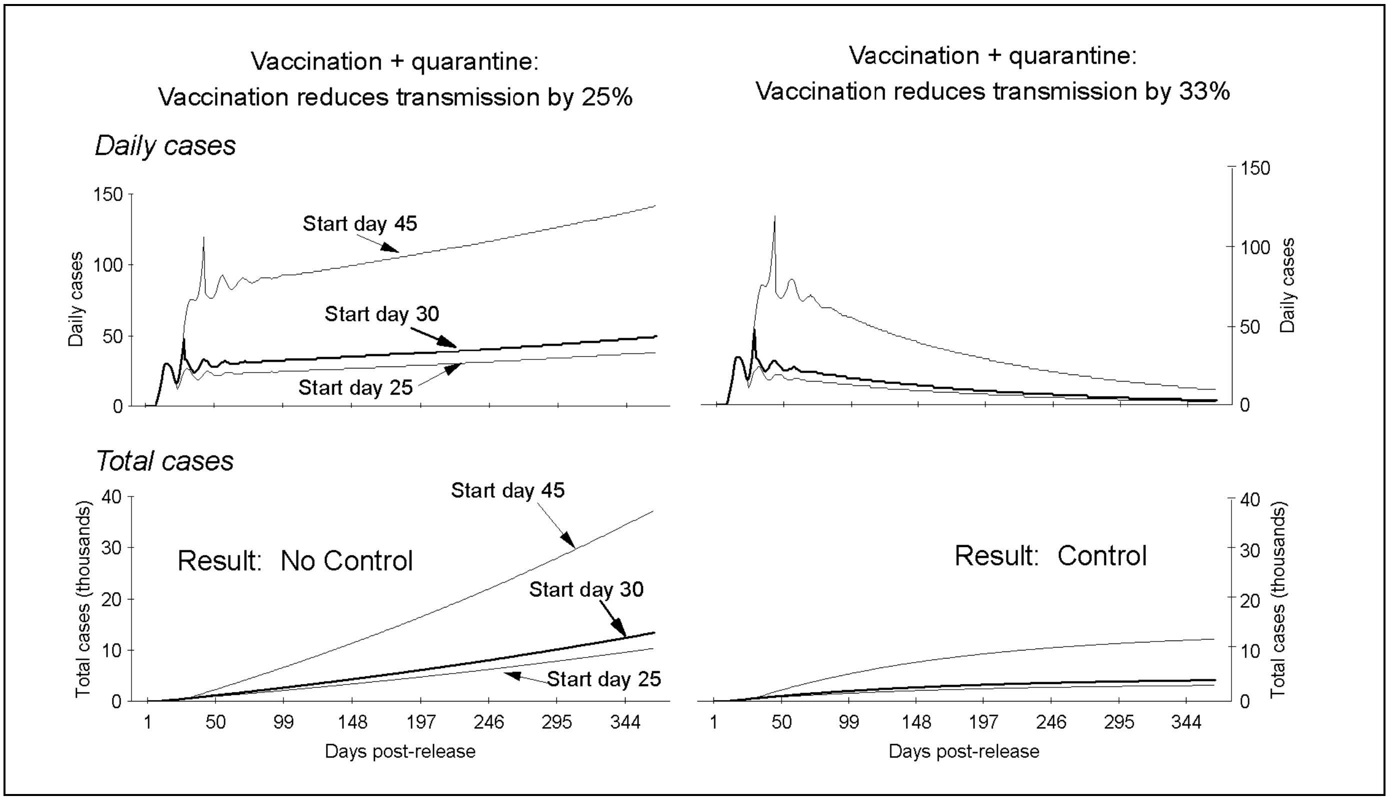

Figure 6. Daily and total cases of smallpox after a combined quarantine (25% daily removal rate) and vaccination campaign for two vaccine-induced reductions in transmission and three postrelease start dates. The graphs show that, when combined with a daily quarantine rate of 25%, vaccination must achieve a $\geq$33% reduction in transmission to stop the outbreak. At a 25% daily removal rate of infectious persons by quarantine, a cohort of all those entering their first day of overt symptoms (i.e., rash) is entirely removed within 17 days (18 to 20 days after incubation) after the first day of overt symptoms, with 90% removed within 9 days. Removal is assumed to start same day as vaccinations. The daily rate of removal by quarantine relates only to the removal of those who are infectious (i.e., are overtly symptomatic). The rate does not include any persons who may be quarantined along with overtly symptomatic patients, such as unvaccinated household contacts. Vaccinating contacts or potential contacts is assumed to result in 25% and 33% reductions in transmission, so that the transmission rate is reduced from 3 to 2.25 and 2 persons infected per infectious person, respectively. Data were generated by assuming 100 initially infected persons and an initial transmission rate of 3 persons infected per infectious person. For clarity, the graphs of daily cases do not include the assumed 100 initially infected persons. The graphs of total cases include those initially infected.

Table 2. Sensitivity analyses: Effect on number of cases of smallpox due to variations in numbers initially infected, numbers infected per infectious person, intervention start days, and quarantine and vaccination effectiveness[a]

| No. initially infected[b] | No. infected per infectious[c] | Start day[d] | Quarantine: % removal per day[e] | Vaccination: % reduction transmission[f] | Impact: Cumulative total at 365 days | Impact: Daily cases at 365 days | Increase or decrease (+/-[g]) |
|---|---|---|---|---|---|---|---|
| Base:100[h] | 3.0 | 30 | 25 | 33 | 4,421 | 3 | - |
| 100 | 2.0 | 30 | 25 | Nil | 2,455 | 2 | - |
| 100 | 2.0 | 30 | 10 | 25 | 10,512 | 2 | - |
| 100 | 2.0 | 45 | 25 | 33 | 1,548 | 0 | - |
| 100 | 5.0 | 30 | 25 | 66 | 4,116 | 0 | - |
| 100 | 5.0 | 45 | 25 | 66 | 19,821 | 1 | - |
| 1,000 | 2.0 | 30 | 10 | 25 | 105,117 | 511 | + |
| 1,000 | 2.0 | 30 | 10 | 33 | 32,125 | 42 | - |

[a]Table 1, Appendix II (see online) is an expanded version of this table.
[b]Number initially infected refers to those who are exposed during a release such that they become infectious. This excludes those who are exposed but either do not become ill or do not become infectious.
[c]The number of persons infected per infectious person is the transmission rate.
[d]Start day, for both quarantine and vaccination interventions, refers to the day postrelease, with the day of release being day 1.
[e]Quarantine refers to removal of infectious persons only, starting on the first day of overt symptoms (i.e., rash). At a 25% daily removal rate, a cohort of all those entering the first day of overt symptoms is entirely removed in 17 days (18 to 20 days postincubation) after day 1 of overt symptoms, with 90% removed in 9 days. At a 10% daily removal, a cohort of all those entering the first day of overt symptoms is entirely removed in 44 days (45 to 47 days post incubation) after day 1 of overt symptoms, with 90% removed in 22 days.
[f]Vaccination is assumed to reduce the transmission rate by a given percentage (e.g., 25% reduction results in transmission declining from 2.0 to 1.5 persons infected per infectious person, and 33% reduces transmission from 2.0 to 1.32).
[g](+) = an increasing rate of daily cases on day 365, and thus the modeled interventions will not stop the transmission of smallpox. (-) = a decreasing rate of daily cases, such that the interventions modeled will eventually stop the transmission of smallpox.
[h] See Figure 6 for complete results related to the base case in the initial modeling scenario.

infected per infectious person (Table 3). If a 25% quarantine-induced daily removal rate is assumed, then vaccination must reduce transmission by 91.5% to 0.26 persons infected per infectious person (Additional results in Appendix II, available at URL: http://www.cdc.gov/ncidod/eid/vol7no6/meltzer_appendix2. htm).

### Vaccinations per Case: Stockpile Issues

We identified 14 outbreaks in which a range of 9 to 102,857 persons were vaccinated per case of smallpox (Table 4). The mean was 14,411 persons vaccinated per case (median 2,155). When fitted to a Gamma probability distribution (35), the 95th, 90th, and 10th percentiles were 7,001, 4,329, and 3.5 doses per case, respectively (Table 4).

In Yugoslavia the number vaccinated per case was approximately 5 times greater than in any other outbreak considered (31). If the Yugoslavia data are removed from the data set (Table 4), the simple average doses per case would be 6,370 (56% decrease), with a median value of 1,801 (16% decrease) doses per case.

If one assumes 4,200 cases result from 100 index cases and a combined quarantine and vaccination program (start day 30: Figure 6), and one uses a median of 2,155 persons vaccinated per case (Table 4), 9,051,000 doses must be made available for use (4,200 x 2,155). The 95th, 90th, and 5th percentiles of this estimate are 29,404,200, 18,181,800, and 14,700, respectively. When the assumed number of persons infected per infectious person is set at 2, the number of cases declines to 1,548 (start on day 45: Table 2), and 3,335,940 vaccine doses must be made available for use (2,155 x 1,548), with 95th, 90th, and 5th percentiles of 10,837,548, 6,701,292, and 5,418, respectively.

### Discussion

The greatest simplification in building our model was the assumption that the supply of susceptible persons was unlimited, so that any specified rate of transmission would be sustained for at least 365 days. In reality, many factors, such as existing immunity and behavior modifications by society (e.g., voluntary or forced quarantine) could limit the supply of susceptible persons, reducing the total number of cases in a 1-year period.

Supply of susceptible persons and assumed rate of transmission are the most important variables influencing the total number of smallpox cases (Tables 1,2). Historically, average transmission rates were well below three persons infected per infectious person (Appendix I, available at URL: http://www.cdc.gov/ncidod/eid/vol7no6/meltzer_appendix1.htm). Variables that can affect the average rate of transmission of smallpox include seasonality, group size, and type of contact ("face-to-face" or "incidental;" Appendix I, Table 5). Our model does not explicitly allow for consideration of such variables, and adjustments to transmission rate resulting from changes in factors such as group size must be done externally to the model.

Another result of assuming an unlimited supply of susceptible persons is that the impact of multiple releases does not "need" to be explicitly modeled. That is, in our model it does not matter if the release initially infects 100 persons who are standing shoulder to shoulder or are each separated by 500 miles. The two variables that can be manipulated to act as proxies for modeling the impact of multiple releases and geographically diverse sites are the transmission rate and the day of the start of interventions. For example, multiple releases may be assumed to result in a lower average transmission rate. Simultaneously, such releases may cause confusion among authorities, the public, and the media, resulting in delay in starting effective interventions. Similarly, releases of smallpox among those perhaps disinclined to interact with authorities (e.g., homeless persons) may go undetected for longer periods of time, also resulting in delayed interventions. We present results from our model of the effect of assuming different transmission rates and start days for an intervention (Tables 1-3). The net result of using these proxy variables to model potential scenarios is that we probably overestimate the spread of disease and the numbers infected. Nonetheless, we feel that the degree of overestimation will probably not substantially affect estimates for thetotalnumberofdosesofvaccinethatshouldbestockpiled.

Another limitation of the model is that it does not explicitly answer the question of how many persons (or what proportion of the population) need to be vaccinated for the transmission rate to decline by, say, 33%. To answer this question, we would need to know two pieces of information:

Table 3. Sensitivity analyses: Minimum levels of intervention needed to stop transmission of smallpox by days 75, 150, and 225 postrelease

| Target stop day[a] | Start day of interventions[a] | Numbers infected per infectious person[b] | Quarantine: Minimum % removal per day[c] | Vaccination: Minimum % reduction in transmission[d] |
|---|---|---|---|---|
| 75 | 30 | 2 | 25.0 | 58.0 (0.84) |
| 75 | 30 | 3 | 25.0 | 76.7 (0.70) |
| 75 | 30 | 5 | 50.0 | 78.9 (1.06) |
| 75 | 45 | 3 | 50.0 | 81.2 (0.57) |
| 150 | 30 | 2 | 25.0 | 25.8 (1.49) |
| 150 | 30 | 3 | 25.0 | 53.7 (1.39) |
| 150 | 30 | 5 | 50.0 | 55.7 (2.22) |
| 150 | 45 | 3 | 50.0 | 33.3 (2.00) |
| 225 | 30 | 2 | 25.0 | 14.3 (1.72) |
| 225 | 30 | 3 | 25.0 | 45.2 (1.65) |
| 225 | 30 | 5 | 50.0 | 46.5 (2.68) |
| 225 | 45 | 3 | 50.0 | 14.8 (2.56) |

See Appendix II, Table 2 (online) for an expanded version of this table.
[a]Target stop day and start day of interventions refer to days postrelease, with day of release being day 1.
[b]The number of persons infected per infectious person is the transmission rate.
[c]Quarantine refers to removal of infectious persons only, starting on the first day of overt symptoms (i.e., rash). Rates are the minimum rates needed, when combined with vaccination, to ensure that there is zero transmission by the target date. At a 25% daily removal rate of infectious persons, a cohort of all those entering their first day of overt symptoms is entirely removed in 17 days (18-20 days postincubation) after day 1 of overt symptoms, with 90% removed in 9 days. With 50% daily removal of infectious persons, a cohort of all those entering the first day of overt symptoms is entirely removed in 7 days (8 to 10 days postincubation) after day 1 of overt symptoms, with 90% removed in 4 days.
[d]Vaccination assumed to reduce the transmission rate by a given percentage (e.g., 25% reduction results in transmission declining from 3.0 to 2.25 persons infected per infectious person). Percentages are the minimum percentage reduction in the assumed rate of transmission needed, when combined with quarantine, to ensure zero transmission by the target date. The resultant transmission rate, after reduction, is in parentheses.

Table 4. Doses of vaccine used to control outbreaks of smallpox: Numbers vaccinated per confirmed case from a variety of outbreaks, 1961-1973

| Site | Year | Population % susceptible | No. of cases | Total vaccinated | Doses used per case | Source |
|---|---|---|---|---|---|---|
| Saiwara village, India | 1968 | 8 | 40 | 1,358[a] | 34 | 27 |
| Nathawala village, India | 1969 | 12 | 12 | 450[b] | 38 | 27 |
| Bawku, Ghana | 1967 | n/a | 66 | 165,449 | 2,507 | 28 |
| Rural Afghanistan | 1969 | n/a[c] | 6 | 508[d] | 85 | 29 |
| Nuatja subdivision, Togo | 1969 | n/a | 6 | 10,818 | 1,803 | 30 |
| Anéono subdivision, Togo | 1969 | 40 | 47 | 294,274 | 6,261 | 30 |
| Yugoslavia | 1972 | n/a | 175 | 18 million | 102,857 | 31 |
| Utinga City, Brazil | 1969 | 57 | 246 | 2,188 | 9 | 32 |
| Botswana | 1973 | 17-27[e] | 30 | 50,000 | 1,667 | 33 |
| London, UK | 1961 | n/a | 3 | 62,000 | 20,667 | 34 |
| West Bromwich, UK | 1961 | n/a | 2 | "limited"[f] | n/a | 34 |
| Bradford, UK | 1961 | n/a | 14 | 250,000 | 17,857 | 34 |
| Birmingham, UK | 1962 | n/a | 1 | "limited"[f] | n/a | 34 |
| Cardiff, UK | 1962 | n/a | 47 | 900,000 | 19,148 | 34 |
| | | | | Mean | 14,411 | |
| | | | | Median | 2,155 | |
| | | | | 95th perct.[g] | 7,001 | |
| | | | | 90th perct.[g] | 4,329 | |
| | | | | 10th perct.[g] | 3.5 | |

[a]This population includes 1,069 revaccinations, accounting for 79% of total vaccinations.
[b]This population includes 323 revaccinations, accounting for 72% of total vaccinations.
[c]The source did not provide population-based estimates of preoutbreak vaccination coverage (as determined by a vaccine scar survey). However, in the four households that contained the six cases, of the 18 family members present at the time of the investigation, 6 (33%) had evidence of preoutbreak vaccination or variolation.
[d]This number excludes some children who had been vaccinated 15 days before the outbreak investigation.
[e]In the sample (n=68,065), susceptibility varied by age. Smallpox vaccination scars were noted among 76% of those $\leq$5 years of age, 83% of those 6 to 14 years of age, and 79% of those $\geq$15 years of age.
[f]The health authorities for West Midlands, which dealt with two of the importations (West Bromwich, Birmingham, UK), limited vaccinations to "...established contacts and medical and ancillary staffs placed at definite risk..." (34). Thus, although the source provides no estimates of the number vaccinated, the description of those targeted for vaccination can lead to the hypothesis that <1,000 persons were vaccinated per case.
[g]The percentiles were calculated by fitting the data to a Gamma distribution (values of parameters: $\alpha = 0.25$; $ß = 58,400$). The chi-square value of the fit of the data to the distribution was 20.57 ($p>0.01$), the Kolmogorov-Smirnov test value was 0.1262 ($p>0.15$), and the Anderson-Darling test statistic was 0.3147 ($p>0.15$).

first, what percentage of the population is truly susceptible to smallpox and could become infectious to others; and second, how would these susceptible persons interact with those infected?[8]

### Vaccination Alone or Combined with Quarantine?

The results from the model demonstrate that it is theoretically possible to completely halt the spread of smallpox by quarantine only (Figure 4; Tables 2,3). The level of quarantine needed, however, may prove impossible to enforce. On the other hand, historically, mass vaccinations alone did not always stop the transmission of smallpox (7,8). Thus, relying solely on either intervention would appear to be unwise, so that a combination of vaccination and quarantine should beused.

Using quarantine has the benefit of lowering the level of effective vaccination needed to stop transmission (Tables 2,3). Furthermore, compared with a vaccination-only intervention, a combined quarantine and vaccination campaign will produce fewer total cases and stop transmission sooner (Table 3). Depending on how vaccination is done, requiring a lower level of effective vaccination could result in fewer vaccinations being administered. Given that the smallpox vaccine occasionally has adverse effects, including death (7,8), any method that reduces the number of vaccinations needed to halt transmission should be examined for possible inclusion into a response plan.

### Doses To Be Stockpiled

The number of estimated doses that must be stockpiled ranges from the 5th percentile estimate of approximately 5,000 doses (assuming approximately 1,500 cases) to a 95th percentile of almost 30 million (assuming approximately 4,200 cases). The latter estimate was generated by assuming an average rate of transmission of three persons infected per infectious person. This assumed level of transmission is well above historical average rates of transmission (Appendix I, available at URL: http://www.cdc.gov/ncidod/eid/vol7no6/meltzer_appendix1.htm). Thus, allowing for factors such as vaccine wastage, stockpiling 40 million doses as recommended by Henderson et al. (5) should be adequate.

[8]Although there are some historical data regarding how infected persons interacted and infected others, all such data were collected when circumstances differed from those of today's societies, particularly with regard to travel and spread of information. Although air and other modes of mass travel were common before smallpox was eradicated, the numbers of travelers and the total miles traveled have vastly increased in the past 30 years. Similarly, although mass media were well known and used in the 1960s and 1970s, more outlets are available to spread information than ever before. It is unknown how these and other changes could affect the spread of smallpox.

Because the pool of smallpox-susceptible persons is now very large, the rate of transmission may be much higher than historical averages, resulting in more cases of smallpox and the need for more vaccine doses stockpiled. For example, if a transmission rate of 5 is assumed and large-scale interventions are started on day 45 postrelease, the 95th percentile of doses that should be stockpiled is 140 million doses (mean 43 million doses; Tables 2,4). Similar estimates are obtained if it is assumed that 1,000 persons are initially infected (Tables 2, 4). Further supporting the argument for stockpiling >40 million doses is the idea that there would be enormous public demand for vaccination in the event of an outbreak.

Stockpiling a large number of doses of smallpox vaccine has three major problems. Building a stockpile of 140 million doses might leave public health officials without needed resources to prepare for and implement other interventions, such as quarantine and public education. Second, a large stockpile poses the problem of deciding how to use it. Investing in such a resource may invite the conclusion that the only suitable response to a deliberate release of smallpox would be a mass vaccination campaign, using as much of the stockpile as possible. An enormous logistical problem would be associated with rapidly vaccinating 140 million persons. Assuming 10 minutes per person vaccinated (excluding patient waiting time), 23 million person-hours would be required to vaccinate 140 million people. In 1947 in New York City it took approximately 1 week to vaccinate 6 million people in response to an outbreak with eight cases (1). An additional problem with trying to mass-immunize >100 million people is that, if a transmission rate of 5 is assumed, disease spread might be so rapid as to “outrun” any mass vaccination attempt (Tables 1,2). The third problem associated with a large stockpile of smallpox vaccine is that a large number of side effects would be generated, including need for treatment with vaccinia immunoglobulin and deaths as a result of adverse reactions (26). Between the demands of vaccination and treatment of side effects, the health-care system would be overburdened, to the detriment of treatment for any other disease or medical emergency.

### Policy Implications

The four most important policy implications from the model results are 1) Delay in intervention will be costly, dramatically increasing the total number of cases; 2) Postrelease intervention should be a combination of quarantine and vaccination; 3) Planning requires not only an appreciation of how many persons may be infected initially, but also an understanding of the likely rate of transmission; and 4) a stockpile of approximately 40 million doses of vaccine should be adequate.

Beyond stockpiling, adequate planning, preparation, and practice must be carried out (36). Such preparation must include training health-care workers to recognize a case of smallpox and what to do if a case is diagnosed. Public health authorities and policymakers need to make detailed plans that fully describe how persons will be quarantined and how quarantine will be enforced. The successful enforcement of quarantine requires political will, public acceptance, and group discipline. Thus, a large part of the preparation for a public health response to smallpox as a bioterrorist weapon must involve educating policymakers and the public as to why quarantine is needed and why relying solely on mass immunizations may not be the magic bullet that some might hope.

Dr. Meltzer is senior health economist, National Center for Infectious Diseases, Centers for Disease Control and Prevention. His research interests focus on assessing the economics of public health interventions such as oral raccoon rabies vaccine, Lyme disease vaccine, influenza vaccination among healthy working adults, and the economics of planning, preparing and practicing for the next influenza pandemic. He uses a variety of research methodologies, including Monte Carlo models, Markov models, contingent valuation (willingness-to-pay) surveys, and nonmonetary units such as Disability Adjusted Life Years.

### References

1. Henderson DA. The looming threat of bioterrorism. Science 1999;283:1279-82.
2. Henderson DA. Smallpox: Clinical and epidemiologic features. Emerg Infect Dis 1999;5:537-9.
3. O'Toole T. Smallpox: An attack scenario. Emerg Infect Dis 1999;5:540-6.
4. Bardi J. Aftermath of a hypothetical smallpox disaster. Emerg Infect Dis 1999;5:547-51.
5. Henderson DA, Inglesby TV, Bartlett JG, Ascher MS, Eitzen E, Jahrling PB, et al. Smallpox as a biological weapon: Medical and public health management. JAMA 1999;281:2127-37.
6. Rao AR. Smallpox. Bombay: The Kothari Book Depot; 1972.
7. Fenner F, Henderson DA, Arita I, Jezek Z, Ladnyi ID. Smallpox and its eradication. Geneva: World Health Organization; 1988.
8. Dixon CW. Smallpox. London: Churchill; 1962.
9. Anderson RM, May RM. Infectious diseases of humans: dynamics and control. New York: Oxford University Press; 1991.
10. Anderson RM, May RM. Population biology of infectious diseases: Part II. Nature 1979;280:455-61.
11. Cliff AD, Haggett P. Statistical modeling of measles and influenza outbreaks. Stat Methods Med Res 1993;2:43-73.
12. Anderson RM, May RM. Population biology of infectious diseases: Part I. Nature 1979;280:361-7.
13. Anderson RM. Transmission dynamics and control of infectious disease agents. In: Anderson RM, May RM, editors. Population biology of infectious diseases. Berlin: Springer-Verlag; 1982. p. 149-77.
14. Aron JL, May RM. The population dynamics of malaria. In: Anderson RM, editor. The population dynamics of infectious diseases: theory and application. London: Chapman and Hall; 1982.
15. Frauenthal JC. Smallpox: When should routine vaccination be discontinued? The UMAP Expository Monograph Series. Boston: Birkhäuser; 1981.
16. Giordano FR, Weir MD, Fox WP. A first course in mathematical modeling. 2nd ed. Pacific Grove, CA: Brooks/Cole Publishing Company; 1997.
17. Christie AR. Infectious diseases: Epidemiology and clinical practice. 3rd ed. New York: Churchill Livingstone; 1980.
18. Singh S. Some aspects of the epidemiology of smallpox in Nepal. Geneva: World Health Organization (WHO/SE/69.10); 1969.
19. Mack TM. Smallpox in Europe, 1950-1971. J Infect Dis 1972;125:161-9.
20. U.S. Bureau of the Census. Statistical abstract of the United States: 1999. 119th ed. Washington: Bureau of the Census; 1999.
21. Royal Commission on Vaccination. A report on vaccination and its results, based on evidence taken by the Royal Commission during the years 1889-1897. Vol 1. The text of the commission report. London: New Sydenham Society; 1898.
22. Smith ADM. Epidemiological patterns in directly transmitted human infections. In: Croll NA, Cross JH, editors. Human ecology and infectious diseases. New York: Academic Press; 1983. p. 333-51.
23. Bartlett MS. Measles periodicity and community size. J Royal Stat Soc Series A 1957;120:48-60.

24. Bartlett MS. Critical community size for measles in the United States. J Royal Stat Soc Series A 1960;123:37-44.
25. Arita I, Wickett J, Fenner F. Impact of population density on immunization programmes. J Hyg Camb 1986;96:459-66.
26. Lane JM, Millar JD. Routine childhood vaccination against smallpox reconsidered. N Engl J Med 1969;281:1220-24.
27. Pattanayak S, Sehgal PN, Raghavan NGS. Outbreaks of smallpox during 1968 in some villages of Jaipur district, Rajasthan. Geneva: World Health Organization (WHO/SE/70.20); 1970.
28. de Sario V. Field investigation of an outbreak of smallpox at Bawku, Ghana: May-October, 1967. Geneva: World Health Organization (WHO/SE/69.24); 1969.
29. Rangaraj AG. An outbreak of smallpox in a village in Afghanistan. Geneva: World Health Organization (WHO/SE/69.9); 1969.
30. Glokpor GF, Agle AN. Epidemiological investigations. Smallpox Eradication Programme in Togo: 1969. Geneva: World Health Organization (WHO/SE/70.21); 1970.
31. Litvinjenko S, Arsic B, Borjanovic S. Epidemiologic aspects of smallpox in Yugoslavia in 1972. Geneva: World Health Organization (WHO/SE/73.57); 1973.
32. de Costa EA, Morris L. Smallpox epidemic in a Brazilian community. Geneva: World Health Organization (WHO/SE/74.64); 1974.
33. Presthus GT, Sibiya JB. A persistent focus of smallpox in Botswana. Geneva: World Health Organization (WHO/SE/74.89); 1974.
34. Great Britain Ministry of Health. Smallpox, 1961-62. Reports on public health and medical subjects, No. 109. London: Her Majesty's Stationery Office; 1963.
35. Evans M, Hastings N, Peacock B. Statistical distributions. 2nd ed. New York: John Wiley & Sons, Inc.; 1993.
36. Kaufmann AF, Meltzer MI, Schmid GP. The economic impact of a bioterrorist attack: Are prevention and postattack intervention programs justifiable? Emerg Infect Dis 1997;3:83-94.
